# Transcriptome analysis reveals the mechanism of mixed oligosaccharides in the response of rice seedlings to abiotic stresses

**DOI:** 10.3389/fpls.2025.1546679

**Published:** 2025-04-28

**Authors:** Yanan Xu, Yigang Yang, Yeran Bai, Makoto Saito, Wei Han, Yuanpei Zhang, Guohua Lv, Jiqing Song, Wenbo Bai

**Affiliations:** ^1^ Institute of Environment and Sustainable Development in Agriculture, Chinese Academy of Agricultural Sciences, Beijing, China; ^2^ China National Rice Research Institute, Hangzhou, China; ^3^ Resonac Corporation (Showa Denko K.K.), Tokyo, Japan; ^4^ Shandong General Station of Agricultural Technology Extension, Jinan, China; ^5^ Institute of Crop Research, Ningxia Academy of Agriculture and Forestry Sciences, Yinchuan, China

**Keywords:** rice seedling, salinity and alkalinity stresses, mixed-oligosaccharides, leaf photosynthesis, physiological mechanism

## Abstract

Salinity and alkalinity stresses severely suppress rice seedling growth and substantially reduce rice yield; whereas the application of oligosaccharides as plant growth regulators has been demonstrated to remarkably enhance crop tolerance to abiotic stresses. To investigate the potential growth-promoting effects of KP-priming (mixed-oligosaccharides, 1.12 mg mL^−1^) on rice seedlings under salinity (100 mmol L^−1^ NaCl) and alkalinity (10 mmol L^−1^ Na_2_CO_3_) stresses, plant morphology and physiology assessments, and transcriptome analyses were performed. The KP-priming significantly improved rice seedling tolerance to salinity and alkalinity stresses, evidenced by increases in plant height, dry matter weight, and fresh weight, and improved root morphology (root length, surface area) and vitality by 10.27–89.06%. Leaf cell membrane stability was improved in KP-priming by increasing the soluble sugar content and superoxide dismutase, peroxidase, and catalase activities by 2.74–97.32%, and reducing accumulation of malondialdehyde and hydrogen peroxide by 17.67–49.70%. KP-priming treatment significantly enhanced leaf photosynthetic capacity through promoting photosynthetic pigments and maximum photochemical efficiency by 2.34–135.76%, and enhancing leaf stomatal aperture by 21.58–75.84%. Transcriptomic analysis revealed that differentially expressed genes in response to KP-priming under salt and alkaline stresses were predominantly associated with photosynthetic pathways. Total 4125 (salinity) and 1971 (alkalinity) DEGs were identified under stresses compared to KP-priming. Transcriptional profiling of KP-priming-treated leaves demonstrated significant up-regulation of key photosynthetic genes, including *OsRBCS5*, *PGR5*, *Se5*, *OsPORA*, *GRA78*, *OsLhcb7*, and *OsPS1-F*. This coordinated gene expression was functionally associated with enhanced leaf photosynthesis capacity and mitigated oxidative damage through improved electron transport and reactive oxygen species scavenging mechanisms. Our findings demonstrated that KP-priming initiated a self-regulatory mechanism in plants, orchestrating a dual protective response that simultaneously mitigated oxidative damage while enhancing photosynthetic efficiency and stress resilience. This study provided initial insights into using KP-priming to alleviate salinity and alkalinity stresses and its underlying molecular mechanisms, which is valuable for both field management practices and understanding rice tolerance to abiotic stresses.

## 1 Introduction

As reported by the Food and Agriculture Organization of the United Nations, soil salinization poses a worldwide challenge, impacting over 100 nations and approximately 1 billion hectares of land, leading to a significant decline in agricultural productivity ranging from 18% to 40% (Wang et al., 2018a; Singh, 2021), which poses a huge challenge to agricultural production and sustainability (Evelin et al., 2019). Salt and alkali stresses are caused by excessive accumulation of neutral salts (e.g. NaCl and Na_2_SO_4_) and alkalinity salts (e.g. NaHCO_3_ and Na_2_CO_3_) in the soil (Yang et al., 2007). Osmotic stress and ionic toxicity cause adverse effects on plants in saline-alkali land (Lu et al., 2022). The area of saline-alkali land in China is about 9.87 × 10^7^ ha, mainly distributed in 17 provinces and regions including Northwest, Northeast, North China, and coastal areas (Li et al., 2005). Additionally, unsuitable farming practices, such as the use of saline water or inadequate drainage systems, have led to a continuous expansion of saline-alkali land. Therefore, by improving the salt-alkali tolerance of crops, it is possible to reclaim degraded land, expand cultivable areas, and enhance food security (Gao et al., 2022).

Rice is an important food crop and sustains almost half of the global population (Wang et al., 2018b). Given China’s large population, maintaining a steady increase in rice production is essential for safeguarding food security. However, rice is highly susceptible to salinity and alkalinity stresses, which cause osmotic stress and physiological drought in the plant when the soil contains excessive inorganic ions. Therefore, a variety of small-molecule organic compounds are synthesized to maintain cellular stability in the plant, such as proline, soluble proteins, and soluble sugars (Sun et al., 2019). The root is the primary organ to perceive salinity and alkalinity stresses, resulting in significant reductions in root number, length, surface area, and dry weight, and severely hinders the capture and utilization of nitrate and ammonium in the soil (Ma et al., 2022). Under normal conditions, reactive oxygen species (ROS) serve as crucial signaling molecules that participate in various physiological and metabolic processes in plants, including growth, development, and responses to environmental stress (Li and Kim, 2022). However, under adverse stresses, ROS accumulation in cells, and excessive superoxide anions (O_2_^·−^) and hydrogen peroxide (H_2_O_2_) cause oxidative damage to cell membranes (Choudhury et al., 2017). This oxidative stress also results in an increase in malondialdehyde (MDA) content (Kang et al., 2021). The antioxidant enzyme system is employed to eliminate excess ROS in the cell and mitigate physiological damage. Enzymes such as superoxide dismutase (SOD), peroxidase (POD), and catalase (CAT) play a crucial role in maintaining ROS homeostasis in the cell. Meanwhile, appropriate ROS levels are essential for normal signaling functions without causing damage to cellular structures (Zhou et al., 2021; Lu et al., 2022). Leaf physiological drought caused by salinity and alkalinity stresses results in insufficient stomatal opening, which in turn reduces transpiration and photosynthetic efficiency (Osakabe et al., 2014). Global transcriptome profiling revealed that the differentially expressed genes (DEGs) in leaves and roots were found between different rice varieties under saline-alkali stress (Zhang et al., 2023). Wang et al. (2023a) have used transcriptomic analyses to reveal that relevant genes, such as *PR5* (*pathogenesis-related protein, Os02g12510*), *FLS2* (*Os04g0349700*), *BRI1* (*protein brassinosteroid-insensitive 1, Os02g0154200*), and *NAC* (*Os02g0285900*), may be the hub genes to participate in promote saline-alkalinity stress tolerance in rice seedlings. The effect of salinity and alkalinity stresses on crops is an extremely complex process involving multiple metabolic processes and physiological mechanisms. Therefore, exploring effective agronomy management techniques has become a hot spot in research on enhancing crop stress tolerance.

Exogenous green ecological regulators play roles in plant nutrient absorption and use efficiency, tolerance to abiotic stress, and better produce quality (Cristofano et al., 2021). In recent years, these regulators have frequently been applied in agricultural fields to enhance stress resistance through regulation of plant growth in consideration of their low toxicity, biodegradability, and high biocompatibility (Wang et al., 2023b; Yang et al., 2024). Oligosaccharides, such as cello-oligosaccharide, xylooligosaccharide, and chitosan oligosaccharide, are novel plant bio-stimulants, distinct from phytohormones, and are small-molecule compounds (**Supplementary Table S1**). They consist of fewer than 10 monosaccharide units linked by glucosidic bonds, are easily soluble in water, and exhibit high biocompatibility (Wu et al., 2020). These have been widely used in forage addition (Del Valle et al., 2018), food production and preservation (Liu et al., 2017; He et al., 2019), and the biomedicine industries (Zhang et al., 2020). Many studies have demonstrated that oligosaccharides, as bio-stimulants, helped plant defense against external stresses by modulating cellular physiological and biochemical responses (Ru et al., 2020; He et al., 2022; Tan et al., 2023). Furthermore, Wang et al. (2023b) and Xu et al. (2024) have shown that applying mixed-oligosaccharides during the seedling stage of cucumber and strawberry can significantly delay natural senescence. These effects were achieved by promoting both root growth and aboveground photosynthetic capacity. In our previous study, we found that pre-sowing treatment of rice seeds with oligosaccharides promoted root radicle and shoot elongation by affecting energy metabolism during the seed germination process, thus effectively alleviating the adverse effects of salt and alkali stress on germination of direct-seeded rice (Yang et al., 2024). However, the impacts of oligosaccharides on morphological characteristics of rice seedlings have not been extensively investigated, especially when subjected to salt and alkali stresses. Moreover, the molecular regulatory pathways governing physiological metabolism in rice seedlings remain poorly understood.

Currently, rice direct-seeded technology is widely used for saving labor and water resources (Lv et al., 2021), which is important for the simplification, specialization, and large-scaling of rice production. The Ningxia Autonomous Region is located in the Yellow River irrigated area, which is one of China’s primary rice-producing regions, and is also the region most severely impacted by soil salinization (Li et al., 2007). Rice is particularly sensitive to saline and alkalinity stress during the seedling stage (Chunthaburee et al., 2016), and improving tolerance of rice seedlings to these stresses through chemical regulatory measures is the most convenient to promote large-scale adoption of direct-seeded rice technology in the saline-alkali region of Western China. Therefore, we investigated the effects of oligosaccharides on the physiological metabolism of rice seedlings under individual saline and alkalinity stresses. Moreover, the expressions of key genes involved in metabolic pathways and multiple physiological mechanisms induced by mixed-oligosaccharides to alleviate stress were identified using transcriptomics. This research aims to address the challenges of rice poor growth caused by saline and alkaline environments, and meet the production demands of direct-seeded rice cultivation. The findings will provide technical support for the efficient production of direct-seeded rice in the saline-alkali regions of Western China and contribute to ensuring regional food security.

## 2 Materials and methods

### 2.1 Plant materials and experiment treatments

Seeds of the typical direct-seeded rice variety Yidao 1 were supplied by the Institute of Crop Research, Ningxia Academy of Agriculture and Forestry Sciences (Yinchuan, Ningxia, China) in the year 2023. Plump rice seeds were selected and sterilized in 5% sodium hypochlorite for 20 min and washed five times with deionized water, then soaked at 25°C with water for 48 h, and germinated at 35°C for 24 h until the radicle broke through the seed coat. Subsequently, germinating rice seeds were planted in a black plastic hydroponic box with dimensions of 127 mm (length) × 87 mm (width) × 114 mm (height). The box contains 96 holes, each with an aperture diameter of 5 mm. The seedlings were cultured in an AR-R850L2 plant growth chamber [XUNON Corporation (Beijing), China] with 60% relative humidity, temperatures of 25/20 ± 1°C (day/night; 12/12 h) with 240 μmol·m^−2^·s^−1^ light intensity, and supplemented with 30% (0–6 days after sowing), 50% (7–14 days after sowing), and 100% (15–28 days after sowing) 1000 x Kimura B nutrient salts Solution (Coolaber, China) replaced every 2 days. The solution detailed contents could be found in **Supplementary Table S2**, pH was maintained in the range of 5.5−5.8.

**Figure 1:**
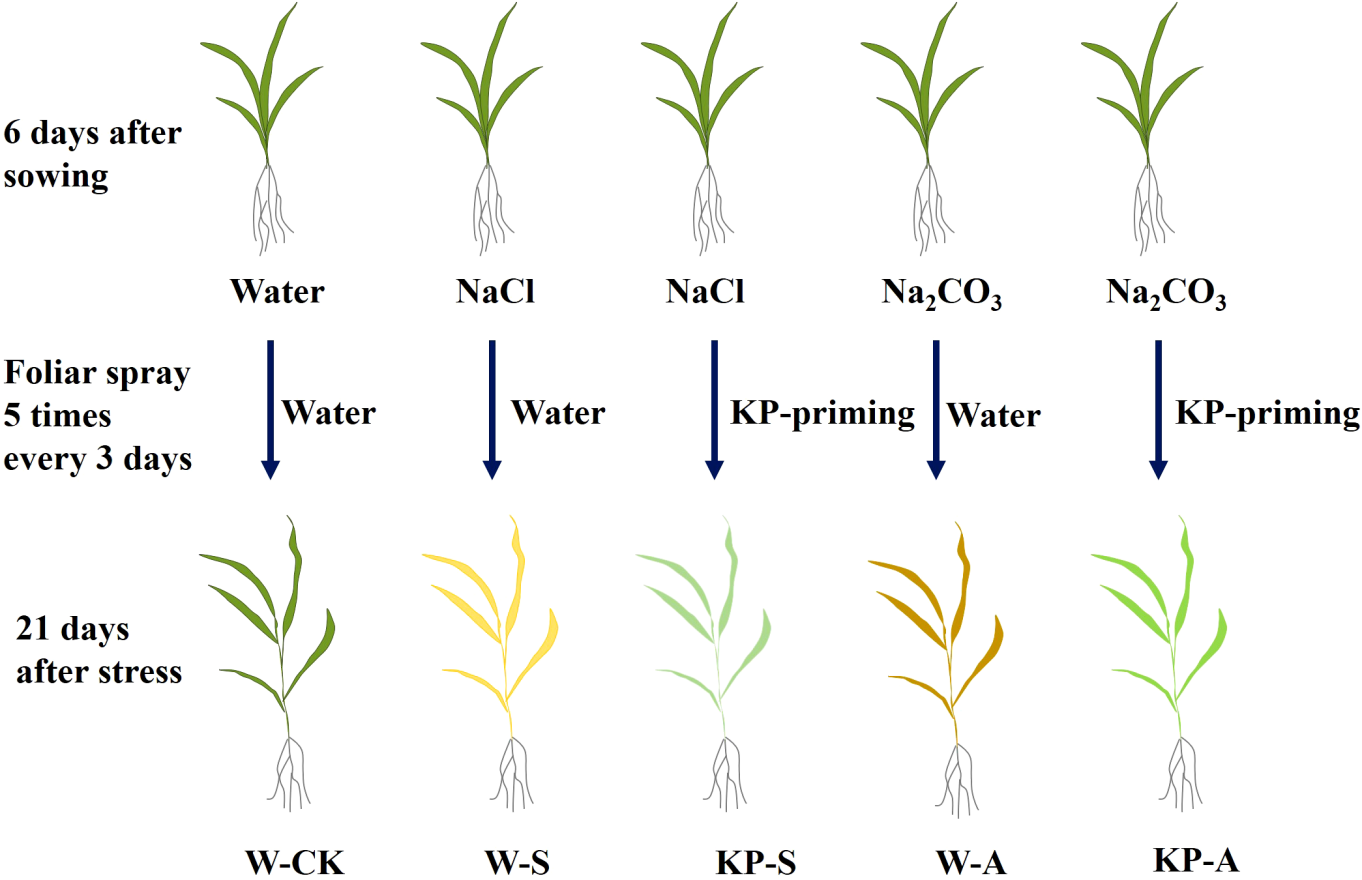
Schematic diagram of the experimental design. After the rice seedlings were respectively exposed to salinity (100 mmol L^−1^ NaCl) and alkalinity (10 mmol L^−1^ Na_2_CO_3_) stresses, they were sprayed with water and KP-priming solution five times every 3 days. These treatments are referred to as W-S, KP-S, W-A, and KP-A, respectively. The control (W-CK) treatment consisted of no stress or KP-priming foliar application.

Based on previous experiments (Yang, 2024), 100 mmol L^−1^ NaCl (salinity stress, S) and 10 mmol L^−1^ Na_2_CO_3_ (alkalinity stress, A) were added into nutrient solution, respectively, to conduct salinity and alkalinity stresses on rice seedlings at 6 days after sowing, with nothing added for the control (CK). Distilled water (W-priming) or 1.12 mg mL^−1^ mixed-oligosaccharides (KROPICO, Resonac Corporation, Japan) solution (KP-priming) were sprayed on seedlings five times (with a 3-day interval) simultaneously. KROPICOKP is a plant growth regulator widely used in facility agriculture in Japan, the proportions of water, KH_2_PO_4_ and oligosaccharides (A, B and C) were 80-99%, 0-12% and 0-10%, respectively. Moreover, A (oligosaccharide), B (chitosan oligosaccharide) and C (cellooligosaccharide) were in a mass ratio of 2:1:1 (https://www.resonac.com/jp/products/chemicals/51/kropico.html). The appropriate concentration of KP-priming was selected based on recommended concentration and previous experiments. Five treatments were conducted (**Figure 1**): distilled water priming + distilled water culture (W-CK), distilled water priming + 100 mmol L^−1^ NaCl culture (W-S), distilled water priming + 10 mmol L^−1^ Na_2_CO_3_ culture (W-A), KP-priming + 100 mmol L^−1^ NaCl (KP-S), and KP-priming + 10 mmol L^−1^ Na_2_CO_3_ culture (KP-A). There were four replicates for each treatment; three replicates were randomly selected from each treatment at 7, 14, and 21 days after stress (DAS) to measure various physiological and biochemical indicators. Two independent experimental replications were conducted to validate the generalizability of the findings.

### 2.2 Determination of seedling morphological characteristics

Intact seedling samples were detached to measure growth characteristics. Aboveground lengths (stem and leaves) were measured using rulers; then they were separated into two parts (aboveground and roots), and immediately weighed to record fresh weight with ME204E analytical balance (Mettler Toledo, USA). Then they were oven-dried at 80°C to obtain dry weight.

Roots were separated from seedlings to wash with tap water. Parts of them were used to determine root activity using the 2,3,5-triphenyltetrazolium chloride method (Li and Zhang, 2016). The rest of the root samples were prepared for root imaging and data analysis using the WinRHIZO root analyzer system (Regent Instruments Inc., Canada).

### 2.3 Determination of chlorophyll content, chlorophyll fluorescence, and stomatal aperture in rice leaves

Chlorophyll (Chl) contents (Chl a and Chl b) were measured according to Lichtenthaler (1987), using a UV-vis 2450/2550 spectrophotometer (Shimadzu, Japan) at two wavelengths (665 and 649 nm). Three fully expanded leaves were detached and two regions were sampled to acquire chlorophyll fluorescence after 30 min of dark adaptation using a fluorescence imaging scheme (Imaging PAM2000, Germany).

Stomatal measurement was performed according to Luo et al. (2022); three fully expanded and similar sized rice leaves were selected from each treatment at 14 DAS. The leaves were placed in solutions containing a mixture of 30 mM KCl and 10 mM MES-Tris (pH 6.15), and exposed to light for 3 h. A light microscope was used to examine the stomata on epidermal strips obtained from leaves. The width, length, perimeter, area, and density of stomatal pores were obtained using Image J software (http://rsbweb.nih.gov/ij).

### 2.4 Determination of soluble sugar, MDA, O_2_^·−^, H_2_O_2_, and antioxidant enzyme activity in rice leaves

Soluble sugar and MDA contents in the leaves were determined according to Li and Zhang (2016). The O_2_ **^·^** ^−^ and H_2_O_2_ accumulation was determined spectrophotometrically following O_2_ **^·^** ^−^–hydroxylamine and H_2_O_2_–titanium peroxide complex formation (Elstner and Heupel, 1976; Rao et al., 1997; Wang et al., 2022). Fresh rice leaves were homogenized in a phosphate-buffered solution over an ice bath (pH 7.8, supplemented with 1.34 mM EDTA-Na^2+^) to finish the antioxidant enzyme assays. Geruisi-Bio assay kits were used to spectrophotometrically determine SOD, POD, and CAT activities according to the manufacturers’ instructions.

### 2.5 RNA-seq library establishment and transcriptome analysis

The total RNA in three replicate samples of rice seedlings leaves at 14 DAS were isolated using the TRIzol method (Invitrogen, CA, USA) (Yang et al., 2023). Sequencing libraries were generated using NEBNext^®^ Ultra™ RNA Library PrepKit for lllumina^®^ (NEB, USA) following manufacturer’s recommendations and index codes were added to attribute sequences to each sample (Peng et al., 2023). The quality of RNA was monitored on 1% agarose gels using an Agilent 2100 Bioanalyzer (Agilent Technologies, CA, USA) and NanoDrop spectrophotometer (Thermo Scientific, DE, USA). The mRNA was extracted from total RNA using poly-T oligo-attached magnetic beads. Fragmentation was performed with divalent cations at an elevated temperature using NEBNext First Strand Synthesis Reaction Buffer (5×). Reverse transcriptase and DNA polymerase were used to synthesize cDNA. Next, the library fragments underwent purification using the AMPure XP system (Beckman Coulter, Beverly, USA) to select appropriate cDNA. Then DNA polymerase and primers were used to obtain PCR products for library preparation, which were sequenced on an Illumina Novaseq 6000 platform by the Beijing Allwegene Technology Company Limited (Beijing, China), and paired-end 150 bp reads were produced.

Differential expression analysis of each pair was performed to determine differentially expressed genes (DEGs), using the DESeq R package (1.10.1). Benjamini and Hochberg’s method was used to adjust P values (P < 0.05). Gene ontology (GO) and Kyoto Encyclopedia of Genes and Genomes (KEGG) enrichment analyses were performed to determine the mechanisms of the DEGs using the GOseq R packages based on Wallenius non-central hyper-geometric distribution (Kanehisa et al., 2008; Young et al., 2010), and KEGG Orthology-based Annotation System software (KOBAS, v2.0), respectively (Mao et al., 2005).

### 2.6 Quantitative real-time PCR analysis

Rice seedling leaves from five treatment groups (W-CK, W-S, W-A, KP-S, KP-A) at 14 DAS were selected for RT-qPCR analysis. The *P* < 0.05 was set as the threshold for identifying differentially-expressed genes. Total RNA was extracted using the TRIzol method (Invitrogen, CA, USA), RNA was reverse transcribed to cDNA using the ReverTra Ace qPCR-RT kit (Toyobo, Osaka, Japan), and the qPCR was performed with SYBR Green Real-Time PCR Master Mix (Toyobo) as previously described (Yang et al., 2023), and three independent RNA samples were prepared for each biological replicate. The rice *Actin1 (Os03g0718100)* gene served as an internal standard to normalize gene expression. The results showed a strong correlation (*r* = 0.86, *P* < 0.01) between the RNA-seq and qPCR data, confirming the reliability of our RNA-seq analysis. Primers used for qPCR are detailed in **Supplementary Table S3**. The relative gene expression levels were calculated using the 2^−ΔΔCt^ method (Schmittgen and Livak, 2008).

### 2.7 Statistical analysis

Data are presented as the means of all replicates. Figures were plotted using Microsoft Excel 2013 and Microsoft Power Point 2013. One-way analyses of variance (ANOVAs) were performed using SAS version 9.4. Duncan’s test was used in ANOVAs to detect significant differences among the mean values for different treatments at P < 0.05.

## 3 Results

### 3.1 KP-priming enhanced rice seedling aboveground growth under abiotic stress

**Figure 2:**
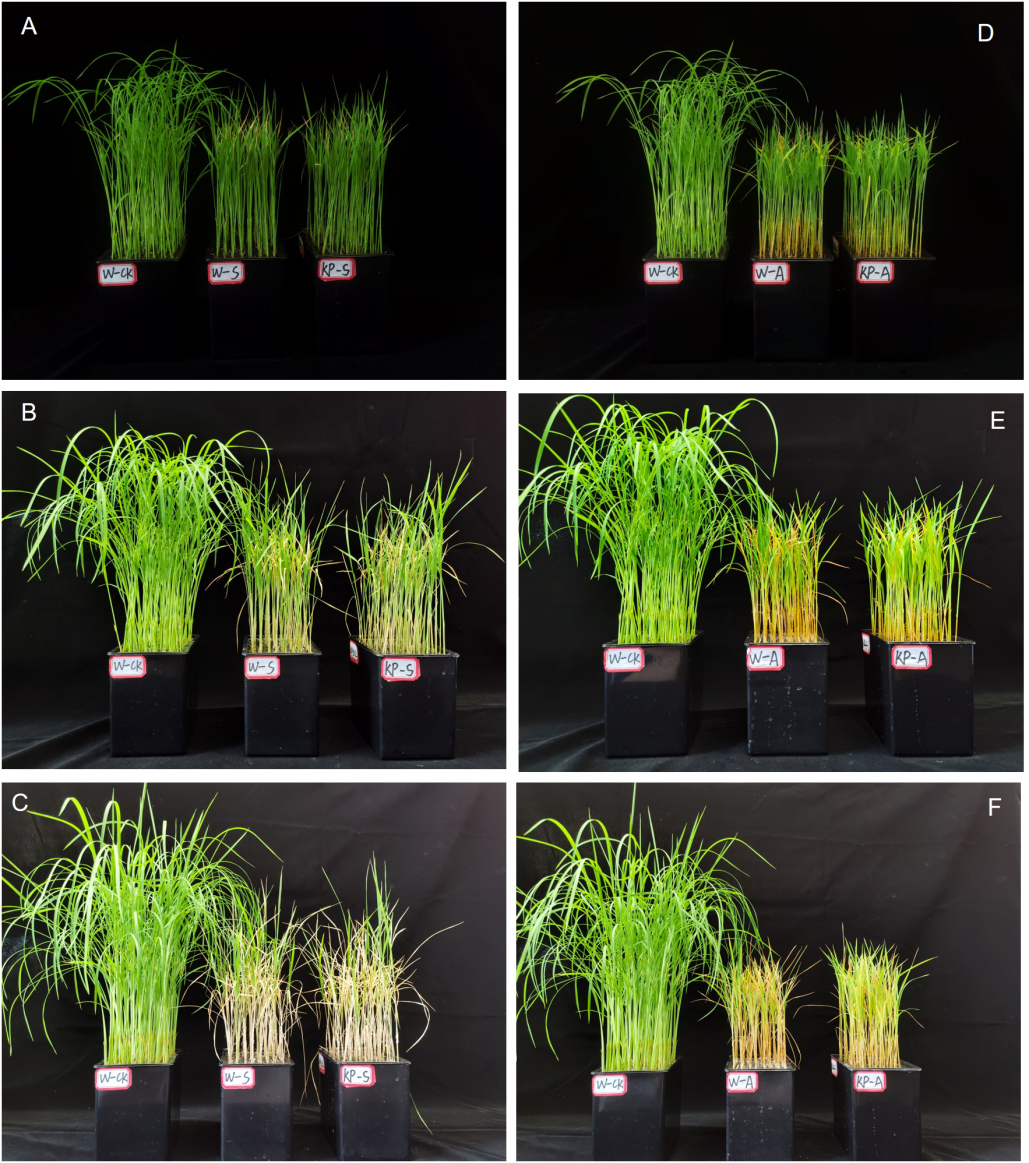
Comparing rice seedling growth condition in water and KP-priming treatment under salinity and alkalinity stress. Rice seedling growth conditions treated with water and KP-priming under salinity stress after 7 d **(A)**, 14 d **(B)**, 21 d **(C)**, respectively; Rice seedling growth conditions treated with water and KP-priming under alkalinity stress after 7 d **(D)**, 14 d **(E)**, 21 d **(F)**, respectively. W-CK, Distilled water priming + distilled water culture; W-S, Distilled water priming + 100 mmol/L NaCl culture; KP-S, KP-priming + 100 mmol/L NaCl culture; W-A, Distilled water priming + 10 mmol/L Na2CO3 culture; KP-A, KP-priming + 10 mmol/L Na2CO3 culture.

**Figure 3:**
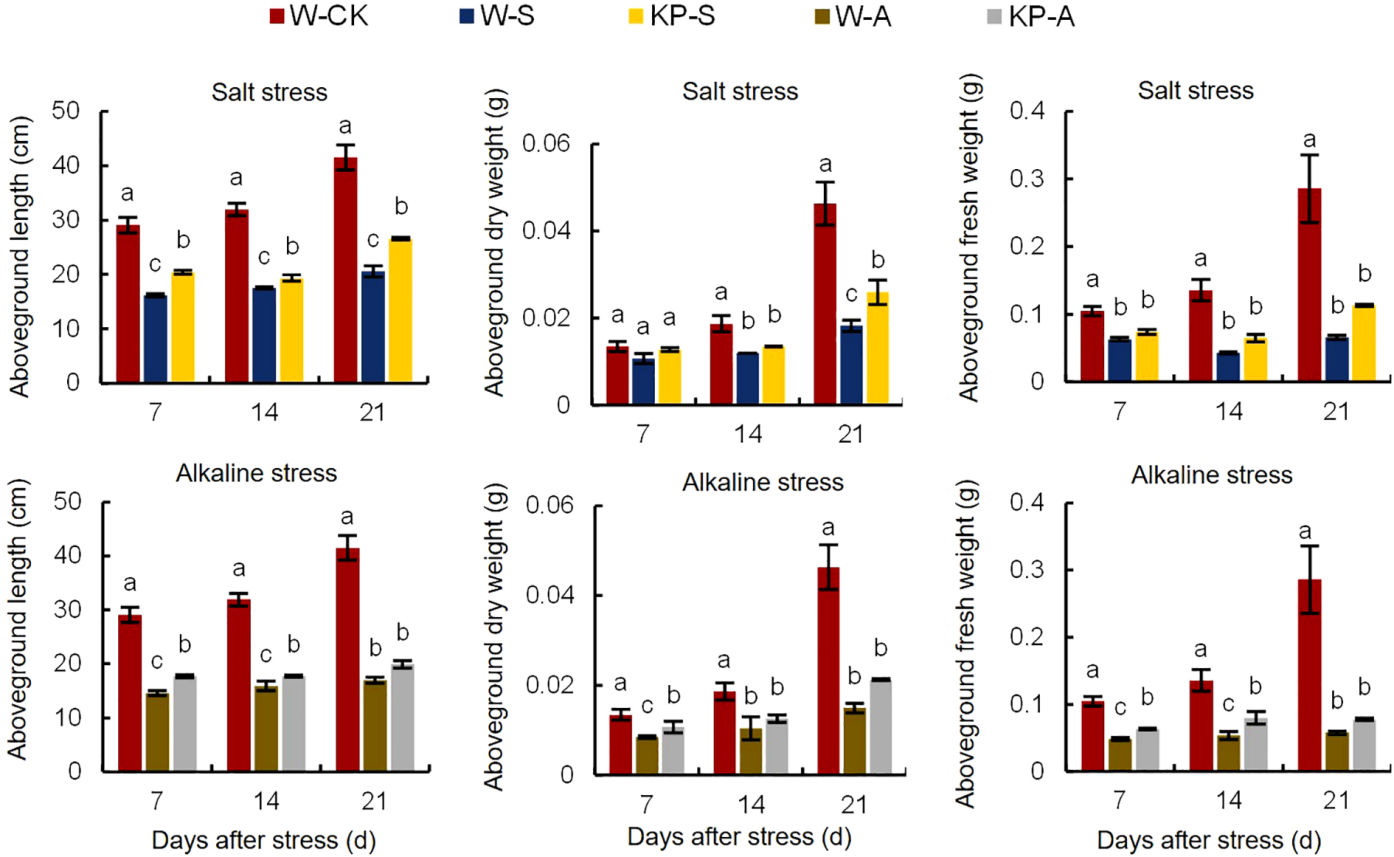
Effects of KP-priming on aboveground length, dry weight, and fresh weight of rice seedlings under salinity and alkalinity stresses. Bars represent ± SE of three replicates. The letters on vertical bars represent significant differences between groups according to Duncan’s multiple range test at *P* < 0.05. W-CK, Distilled water priming + distilled water culture; W-S, Distilled water priming + 100 mmol/L NaCl culture; KP-S, KP-priming + 100 mmol/L NaCl culture; W-A, Distilled water priming + 10 mmol/L Na_2_CO_3_ culture; KP-A, KP-priming + 10 mmol/L Na_2_CO_3_ culture.

At 7 DAS, W-S and W-A treatment significantly hindered aboveground growth of rice seedlings, resulting in reduced plant height and noticeable leaf yellowing, when compared to the W-CK treatment (**Figure 2**). Compared with 7 DAS, the growth condition of rice seedlings treated with salinity and alkalinity stresses showed greater difference to W-CK at 21 DAS, indicating that the effects of stress on rice seedling growth were aggravated with stress time. Compared to W-CK, salinity (W-S) and alkalinity (W-A) stress significantly reduced plant height and aboveground fresh and dry weights of rice seedlings at each stage by 44.44–59.04%, 39.69–79.78%, and 35.71–67.77%, respectively (**Figure 3**). Compared to W-S and W-A, the growth of rice seedlings under KP-S and KP-A was significantly improved. Compared to W-priming, KP-priming significantly increased plant height by 10.27–29.34% (7–21 DAS) and aboveground dry weight of rice seedlings by 42.41% (21 DAS) under salinity stress, respectively. Similarly, under alkalinity stress KP-priming significantly increased the plant height by 11.53–21.46% (7–14 DAS), above-ground fresh weight by 30.27–48.70% (7–14 DAS) and dry weight of rice seedlings by 26.77% (21 DAS), respectively. These results indicated that KP-priming had distinct regulatory effects on aboveground growth of seedlings under different stress environments. During 7–21 DAS, KP-priming progressively enhanced the aboveground growth of seedlings under both salinity and alkalinity stresses as stress duration and spraying frequency increased. Specifically, the positive effects of KP-priming on plant height increased from 26.19% (7 DAS) to 29.34% (21 DAS) under salinity stress; and the promotion effects of KP-priming on aboveground fresh weight increased from 30.27% (7 DAS) to 48.70% (14 DAS) under alkalinity stress.

### 3.2 KP-priming enhanced rice seedling underground growth under abiotic stress

Compared to W-CK, W-S and W-A treatments severely inhibited the root growth of rice seedlings (**Table 1**). Root length, fresh weight, dry weight, surface area, diameter, volume, and activity were significantly reduced by 8.75–56.29% for the W-S treatment and 14.60–46.33% for the W-A treatment during the period 7–21 DAS, respectively. The KP-priming significantly alleviated the inhibition of root growth under salinity and alkalinity stress, promoting root dry weight, length, surface area, and activity compared to stress treatments. Furthermore, the enhancing effects of KP-priming on root development progressively intensified with prolonged stress duration and increased spraying frequency. Thus, the positive effects of KP-priming in salinity stress on root length and surface area increased from 25.89% (7 DAS) and 17.70% (7 DAS) to 40.37% (21 DAS) and 22.54% (14 DAS), respectively. Additionally, root activity increased from 28.70% (14 DAS) to 31.30% (21 DAS) compared to W-priming. Similarly, under alkalinity stress, KP-priming increased root dry weight from 35.62% (7 DAS) to 89.06% (14 DAS) compared to W-priming.

**Table 1:** Effects of KP-priming on rice seedling root length, fresh weight, dry weight, surface area, average diameter, volume, and activity under salinity and alkalinity stresses.

Date	Treatment	Root length (cm)	Root fresh weight (g)	Root dry weight (g)	Root surface area (cm^2^)	Root average diameter (mm)	Root volume (cm^3^)	Root activity (μg h^−1^ g^−1^)
7 DAS	W-CK	10.32 ± 0.97aA	0.091 ± 0.004aA	0.0040 ± 0.0002aA	6.89 ± 0.17aA	0.21 ± 0.02aA	0.037 ± 0.005aA	53.55 ± 1.81aA
	W-S	6.39 ± 0.29c	0.066 ± 0.004b	0.0030 ± 0.0003b	4.80 ± 0.16c	0.23 ± 0.01a	0.030 ± 0.002b	45.74 ± 3.62a
	KP-S	8.04 ± 0.39b	0.064 ± 0.004b	0.0036 ± 0.0002a	5.64 ± 0.36b	0.22 ± 0.01a	0.029 ± 0.002b	50.74 ± 1.65a
	W-A	6.15 ± 0.16C	0.078 ± 0.002A	0.0024 ± 0.0003B	5.69 ± 0.22B	0.25 ± 0.01A	0.035 ± 0.001A	45.74 ± 1.37C
	KP-A	7.44 ± 0.30B	0.076 ± 0.008A	0.0033 ± 0.0003A	5.60 ± 0.31B	0.24 ± 0.01A	0.031 ± 0.004A	49.80 ± 4.21B
14 DAS	W-CK	11.26 ± 0.27aA	0.076 ± 0.005aB	0.0028 ± 0.0004aB	6.98 ± 0.70aA	0.20 ± 0.01bB	0.032 ± 0.005aB	38.89 ± 3.52bA
	W-S	6.68 ± 0.34c	0.064 ± 0.001a	0.0023 ± 0.0004a	4.73 ± 0.18c	0.23 ± 0.00a	0.029 ± 0.003a	36.94 ± 2.70b
	KP-S	8.81 ± 0.16b	0.072 ± 0.005a	0.0026 ± 0.0001a	5.80 ± 0.09b	0.23 ± 0.01a	0.030 ± 0.003a	47.55 ± 1.26a
	W-A	7.58 ± 0.36C	0.100 ± 0.008A	0.0021 ± 0.0004B	5.64 ± 0.38B	0.24 ± 0.01A	0.035 ± 0.004AB	27.25 ± 0.25B
	KP-A	8.74 ± 0.24B	0.110 ± 0.014A	0.0040 ± 0.0004A	6.62 ± 0.15A	0.26 ± 0.02A	0.044 ± 0.002A	46.84 ± 4.44A
21 DAS	W-CK	13.89 ± 1.46aA	0.149 ± 0.021aA	0.0056 ± 0.0008aA	10.96 ± 1.15aA	0.21 ± 0.01aB	0.046 ± 0.016aA	51.27 ± 1.81aA
	W-S	7.49 ± 0.18c	0.065 ± 0.007b	0.0024 ± 0.0004b	5.21 ± 0.26b	0.19 ± 0.00b	0.025 ± 0.001a	28.09 ± 3.14c
	KP-S	10.52 ± 0.42b	0.084 ± 0.01b	0.0038 ± 0.0004b	6.07 ± 0.09b	0.18 ± 0.01b	0.028 ± 0.001a	36.88 ± 1.39b
	W-A	7.46 ± 0.18B	0.102 ± 0.008B	0.0041 ± 0.0005A	6.61 ± 0.25B	0.27 ± 0.03A	0.042 ± 0.003A	40.10 ± 2.17B
	KP-A	9.56 ± 0.39B	0.108 ± 0.005B	0.0047 ± 0.0002A	6.55 ± 0.28B	0.28 ± 0.01A	0.046 ± 0.001A	51.44 ± 0.88A

Lowercase letters represent significant differences among W-CK, W-S, and KP-S treatments; uppercase letters represent significant differences among W-CK, W-A, and KP-A treatments. W-CK, Distilled water priming + distilled water culture; W-S, Distilled water priming + 100 mmol/L NaCl culture; KP-S, KP-priming + 100 mmol/L NaCl culture; W-A, Distilled water priming + 10 mmol/L Na_2_CO_3_ culture; KP-A, KP-priming + 10 mmol/L Na_2_CO_3_ culture.

### 3.3 KP-priming enhanced rice seedling leaf cell membrane stability under abiotic stress

**Figure 4:**
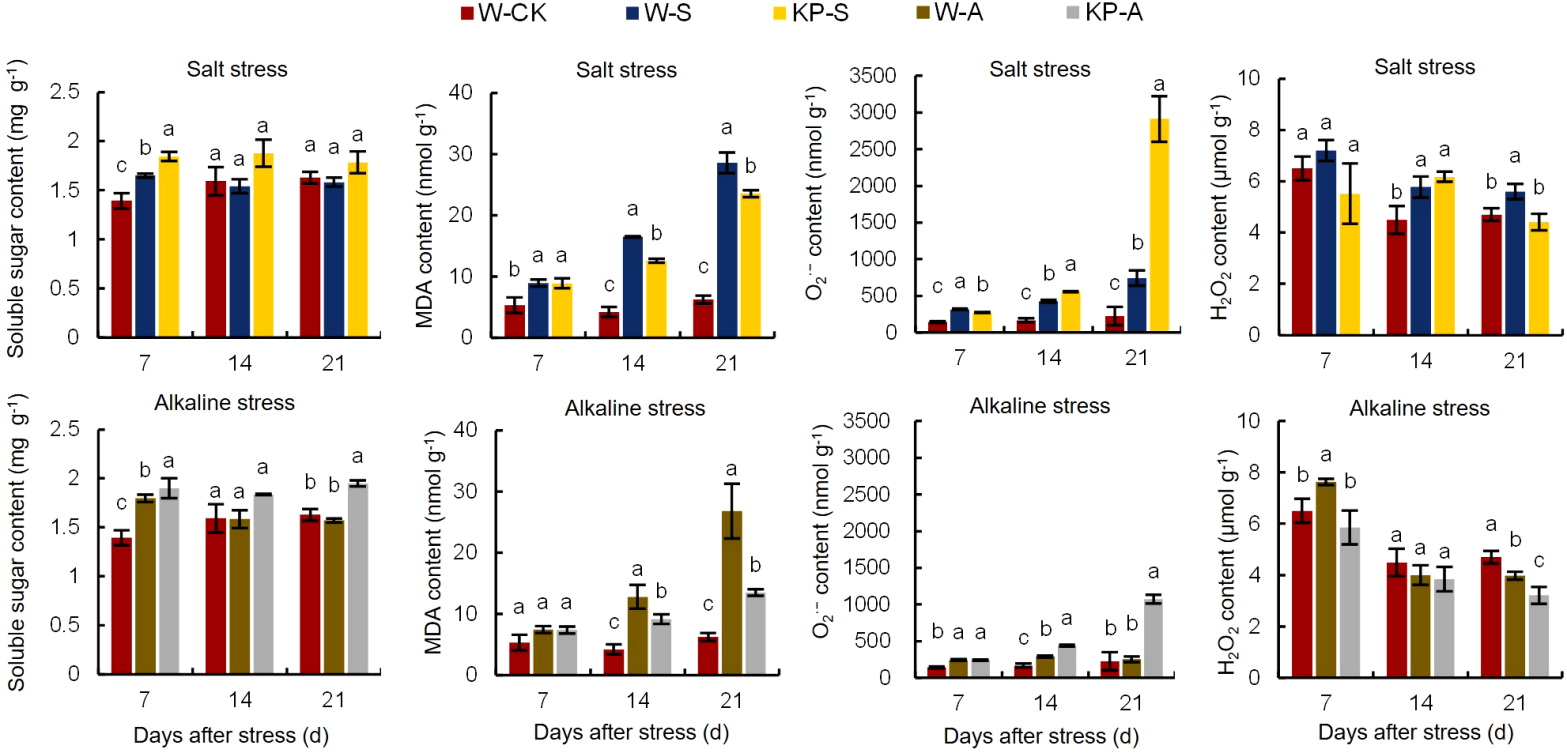
Effects of KP-priming on soluble sugar, MDA, O_2_^·−^, and H_2_O_2_ content in rice seedlings under salinity and alkalinity stresses. Bars represent ± SE of three replicates. The letters on vertical bars represent significant differences between groups according to Duncan’s multiple range test at *P* < 0.05. W-CK, Distilled water priming + distilled water culture; W-S, Distilled water priming + 100 mmol/L NaCl culture; KP-S, KP-priming + 100 mmol/L NaCl culture; W-A, Distilled water priming + 10 mmol/L Na_2_CO_3_ culture; KP-A, KP-priming + 10 mmol/L Na_2_CO_3_ culture.

The W-S treatment led to an 18.45% reduction in the soluble sugar content of seedling leaves at 7 DAS compared to W-CK, whereas KP-S resulted in a significant increase of 11.76% compared to W-S (**Figure 4**). Similarly, W-A treatment caused a 29.05% decrease in soluble sugar content at 7 DAS compared to W-CK, while KP-A demonstrated a significant increase of 5.70% compared to W-A. Salinity and alkalinity stresses significantly increased the MDA content in seedling leaves compared to W-CK: by 67.51–358.62% (7–21 DAS) in W-S and 205.13–330.17% (14–21 DAS) in W-A. The leaf MDA contents in the KP-S and KP-A treatments were significantly decreased compared to stress treatments, with the inhibitory effect on MDA of KP-A increasing from 28.57% (14 DAS) to 49.70% (21 DAS). This indicated that the inhibitory effect of KP-priming on MDA gradually intensified with longer stress duration and higher spraying frequency.

Salinity and alkalinity stresses significantly increased the accumulation of O_2_ **^·^** ^−^ and H_2_O_2_ in leaves (**Figure 4**). Compared to W-CK, the O_2_ **^·^** ^−^ and H_2_O_2_ contents in the W-S treatment were significantly increased by 120.61–225.34% (7–14 DAS) and 19.06–28.52% (14–21 DAS), and for W-A were significantly increased by 71.23–73.80% (7–14 DAS) and 17.34% (14 DAS), respectively. The KP-priming significantly increased O_2_ **^·^** ^−^ accumulation in rice seedling leaves after stress, with the effects becoming more pronounced as the stress duration increase. The enhancement of O_2_ **^·^** ^−^ accumulation in KP-S increased from 29.91% (7 DAS) to 291.75% (21 DAS), while in KP-A increased from 49.93% (7 DAS) to 324.34% (21 DAS), respectively. However, KP-priming significantly decreased H_2_O_2_ content by 21.26% (21 DAS) in salinity stress and 19.20% (21 DAS) in alkalinity stress.

**Figure 5:**
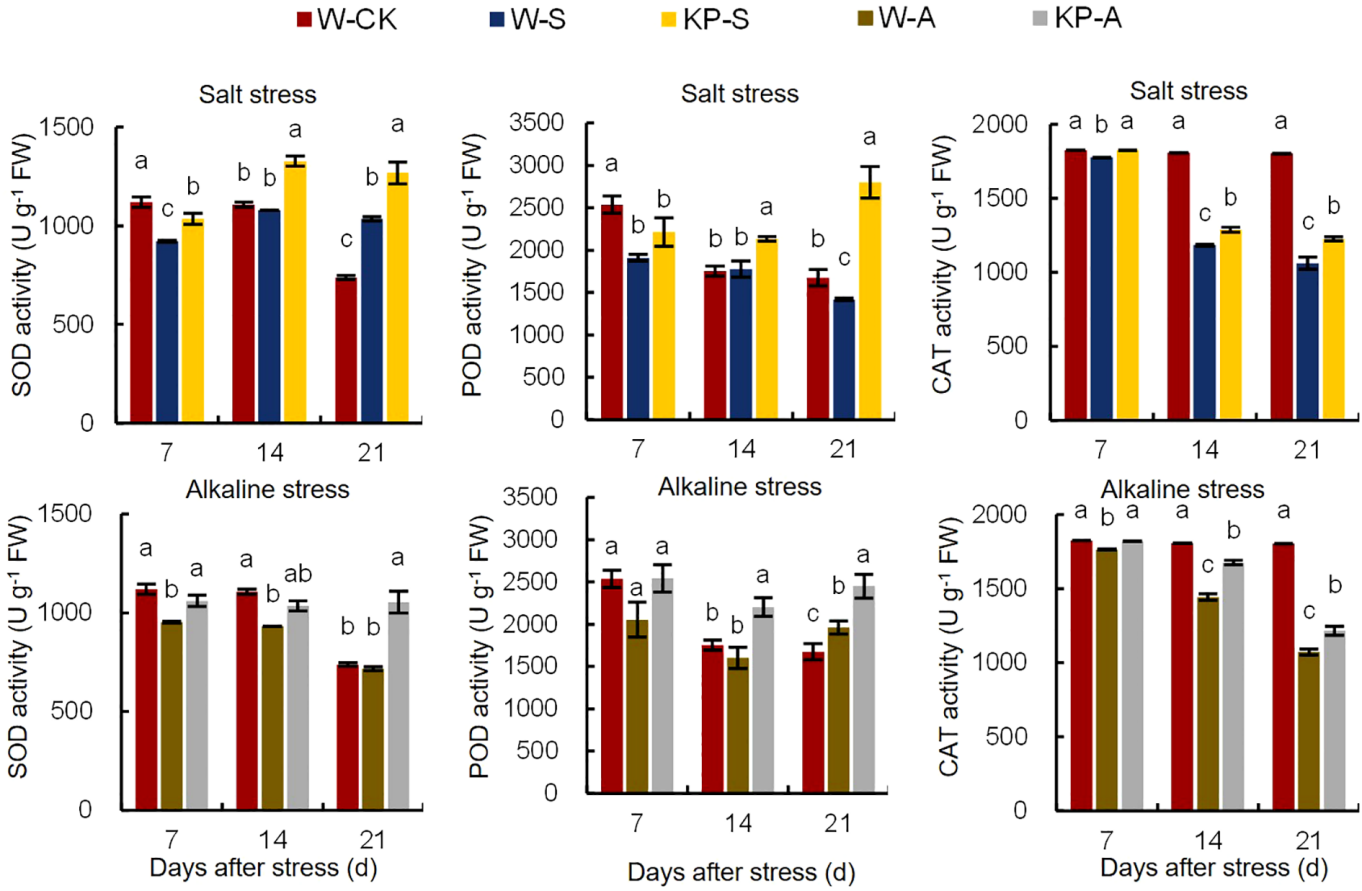
Effects of KP-priming on SOD, POD, and CAT activities in rice seedlings under salinity and alkalinity stresses. Bars represent ± SE of three replicates. The letters on vertical bars represent significant differences between groups according to Duncan’s multiple range test at *P* < 0.05. W-CK, Distilled water priming + distilled water culture; W-S, Distilled water priming + 100 mmol/L NaCl culture; KP-S, KP-priming + 100 mmol/L NaCl culture; W-A, Distilled water priming + 10 mmol/L Na_2_CO_3_ culture; KP-A, KP-priming + 10 mmol/L Na_2_CO_3_ culture.

Abiotic stress causes excessive accumulation of ROS in cells, disrupting normal physiological processes. However, the antioxidant system plays a crucial role in reducing ROS levels and maintaining cellular stability. The SOD, POD, and CAT activities were significantly reduced by 17.61% (7 DAS), 15.17–24.76% (7 and 21 DAS), and 2.61–41.17% (7–21 DAS) in W-S compared to W-CK, respectively, while only SOD and CAT activities were significantly reduced by 14.90–15.83% (7–14 DAS) and 3.39–40.60% (7–21 DAS) in W-A, respectively (**Figure 5**). The KP-priming induced significant increases in antioxidant enzyme activities in leaves compared to W-priming, with SOD, POD, and CAT activities under salinity stress raised by 12.25–22.92% (7–21 DAS), 20.17–97.32% (14–21 DAS), and 2.74–15.52% (7–21 DAS), respectively. In addition, KP-priming significantly enhanced SOD, POD, and CAT activities under alkalinity stress by 11.34–46.93% (7 and 21 DAS), 24.94–37.55% (14 and 21 DAS) and 3.19–16.17% (7–21 DAS), respectively.

### 3.4 KP-priming enhanced rice seedling leaf photosynthetic capacity under abiotic stress

**Figure 6:**
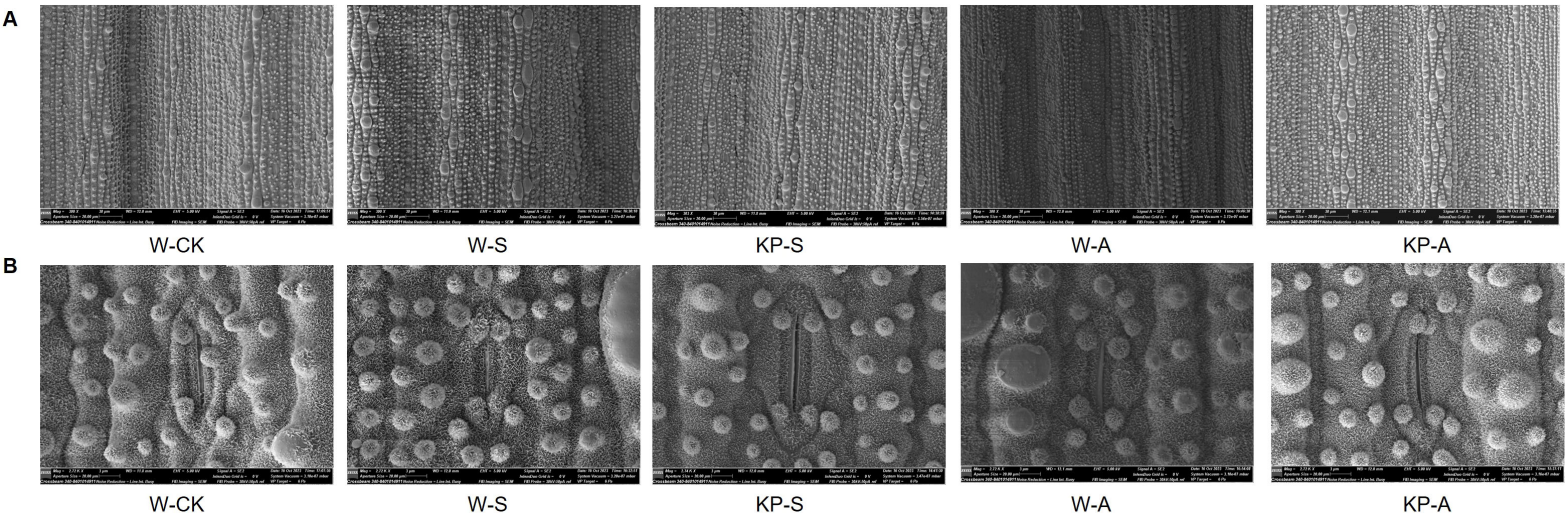
Effects of KP-priming on stomata structure of rice seedling leaves under salinity and alkalinity stresses. Multiple stomata visualization of the back of rice leaves in different treatments at 14 d after stress (**A**, Bar = 20 µm); single stomata visualization of the back of rice leaves in different treatments at 14 d after stress (**B**, Bar = 3 µm). W-CK, Distilled water priming + distilled water culture; W-S, Distilled water priming + 100 mmol/L NaCl culture; KP-S, KP-priming + 100 mmol/L NaCl culture; W-A, Distilled water priming + 10 mmol/L Na_2_CO_3_ culture; KP-A, KP-priming + 10 mmol/L Na_2_CO_3_ culture.

Stomata play a crucial role in maintaining cell osmotic pressure and regulating the water retention capacity of leaves. They also serve as an essential channel for gas exchange to enhance leaf photosynthetic capacity. Microscopy of stomatal structure of seedling leaves at 14 DAS showed that the stresses significantly reduced stomatal uniformity and size (**Figure 6**). Moreover, stomatal length was significantly reduced by 23.13% in W-S compared to W-CK, and stomatal length, perimeter, and density in W-A were reduced by 16.11%, 12.68%, and 33.33%, respectively. The KP-priming significantly alleviated the effects of stress on stomatal closure, with KP-S showing higher stomatal length, width, perimeter, and area than W-S by 39.49%, 26.93%, 34.45%, and 75.84%, respectively. The KP-A showed higher stomatal length, perimeter, and density than W-A by 27.72%, 21.58%, and 53.13%, respectively (**Table 2**).

**Table 2:** Effects of KP-priming on stomata morphological characteristics of rice seedling leaves under salinity and alkalinity stresses.

Treatment	Length (μm)	Width (μm)	Perimeter (μm)	Area (μm^2^)	Density (mm^−2^)
W-CK	21.62 ± 0.59aA	10.96 ± 0.75bA	65.15 ± 2.57abA	237.14 ± 22.20bA	319.46 ± 52.83aA
W-S	16.62 ± 1.97b	11.15 ± 1.22b	55.54 ± 6.07b	186.60 ± 39.40b	209.64 ± 51.87a
KP-S	23.18 ± 0.42a	14.15 ± 0.98a	74.67 ± 2.11a	328.12 ± 23.33a	266.21 ± 15.25a
W-A	18.14 ± 1.55B	10.31 ± 1.14A	56.89 ± 4.65B	187.55 ± 32.34A	212.97 ± 25.12B
KP-A	23.16 ± 0.77A	11.42 ± 0.83A	69.17 ± 3.17A	264.99 ± 28.17A	326.11 ± 40.35A

Lowercase letters represent significant differences among W-CK, W-S, and KP-S treatments; uppercase letters represent significant differences among W-CK, W-A, and KP-A treatments. W-CK, Distilled water priming + distilled water culture; W-S, Distilled water priming + 100 mmol/L NaCl culture; KP-S, KP-priming + 100 mmol/L NaCl culture; W-A, Distilled water priming + 10 mmol/L Na_2_CO_3_ culture; KP-A, KP-priming + 10 mmol/L Na_2_CO_3_ culture.

**Figure 7:**
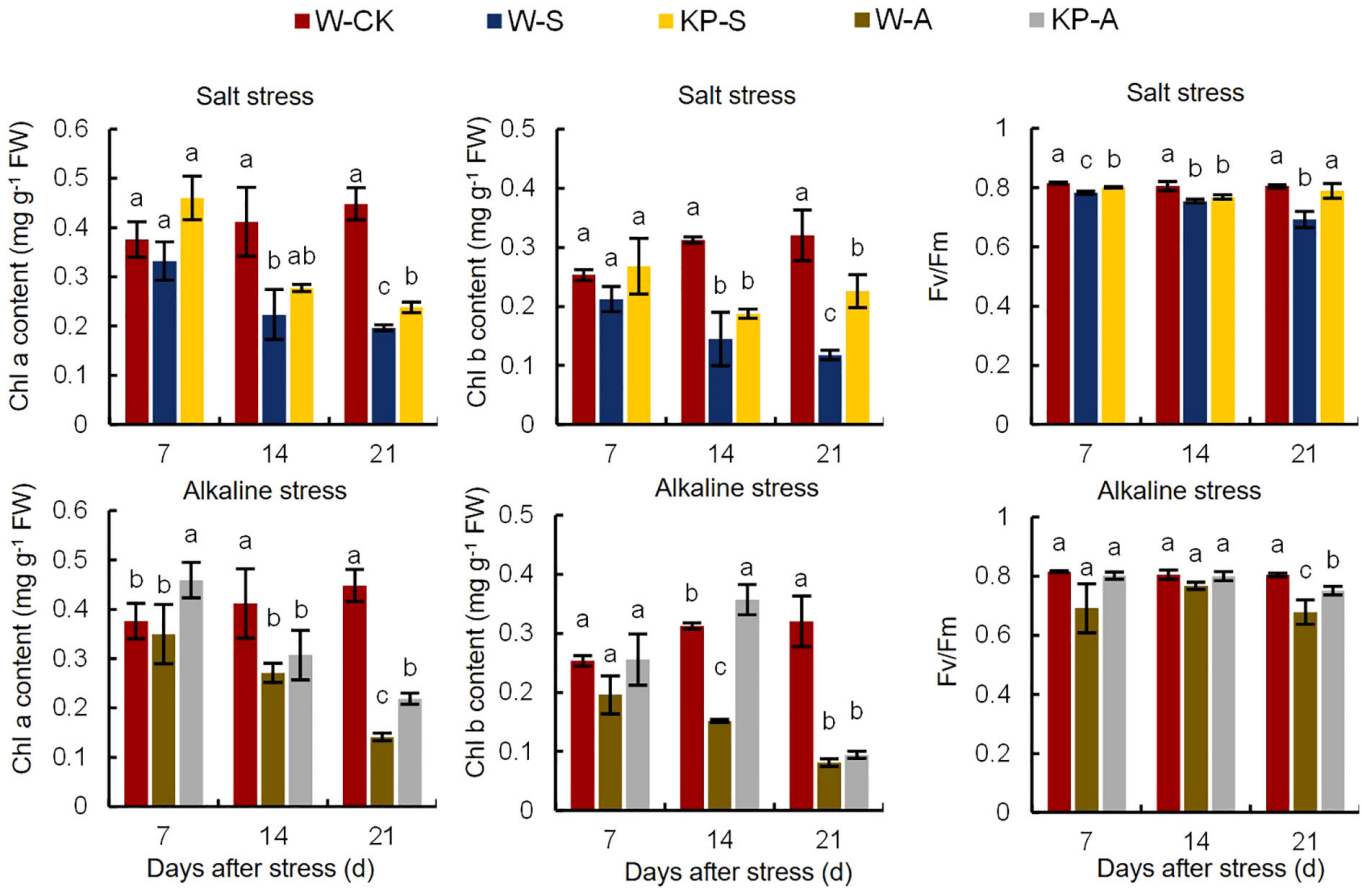
Effects of KP-priming on chlorophyll a/b content and Fv/Fm of rice seedling leaves under salinity and alkalinity stresses. The bars represent ± SE of three replicates. The letters on vertical bars represent significant differences between groups according to Duncan’s multiple range test at *P* < 0.05. W-CK, Distilled water priming + distilled water culture; W-S, Distilled water priming + 100 mmol/L NaCl culture; KP-S, KP-priming + 100 mmol/L NaCl culture; W-A, Distilled water priming + 10 mmol/L Na_2_CO_3_ culture; KP-A, KP-priming + 10 mmol/L Na_2_CO_3_ culture.

The Chl a and Chl b are important components of photosynthetic pigments involved in capturing and converting light energy in photosynthesis. Rice seedlings treated with salinity and alkalinity stresses showed significant leaf yellowing (**Figure 1**). Compared to W-CK, Chl a content was significantly reduced by 11.82–56.20% (7–21 DAS) in W-S and by 34.09–68.51% (14–21 DAS) in W-A; correspondingly Chl b content was significantly reduced by 53.70–63.32% (14–21 DAS) and 51.56–74.71% (14–21 DAS). Furthermore, the decline in pigment content became more evident as stress duration increased (**Figure 7**). KP-priming significantly elevated the Chl a and Chl b contents in seedling leaves under salinity stress compared to W-priming, with increases of 21.86-38.79% (at 7 and 21 DAS) and 92.11% (at 21 DAS), respectively. Similarly, under alkalinity stress, KP-priming enhanced Chl a and Chl b contents by 31.33–55.20% (7 and 21 DAS) and 135.76% (14 DAS),respectively.

Chlorophyll fluorescence Fv/Fm is an important parameter for assessing the photosynthetic physiology of crops under stress conditions. Compared to W-CK, Fv/Fm was significantly reduced by 4.04–13.99% (7–21 DAS) in W-S and by 15.69% (21 DAS) in W-A. The reduction in Fv/Fm was pronounced with longer stress exposure. KP-priming significantly alleviated the negative effects of stress on leaf photosynthesis, with the enhancement in leaf photosynthetic capacity gradually increasing over time. Under salinity stress, KP-priming increased Fv/Fm by 2.34% (7 DAS) and 14.00% (21 DAS) compared to W-priming, and by 10.65% (21 DAS) under alkalinity stress (**Figure 7**).

### 3.5 Global expression analysis of rice seedling leaves when treated with KP-priming

#### 3.5.1 Identification of DEGs

**Figure 8:**
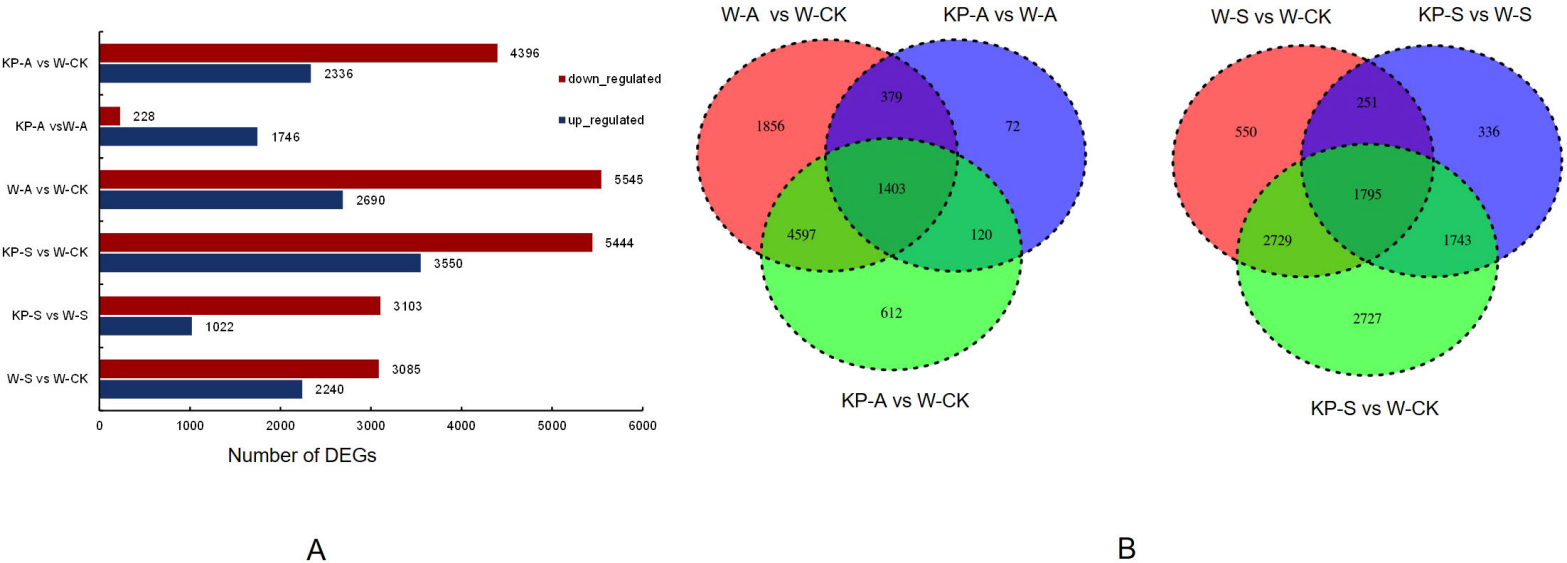
Transcriptome analysis of rice seedling leaves in response to different treatments. Number of differentially expressed genes (DEGs) in different treatments **(A)**; Venn diagram of DEGs in different treatments **(B)**. W-CK, Distilled water priming + distilled water culture; W-S, Distilled water priming + 100 mmol/L NaCl culture; KP-S, KP-priming + 100 mmol/L NaCl culture; W-A, Distilled water priming + 10 mmol/L Na_2_CO_3_ culture; KP-A, KP-priming + 10 mmol/L Na_2_CO_3_ culture.

To investigate the molecular mechanisms underlying the regulation of leaf photosynthetic capacity by KP-priming, transcriptome analysis was conducted on rice seedling leaves at 14 DAS (**Figure 8**). The PCA, volcano plots, and heat map images, etc., were shown in **Supplementary Figure S1** and **Supplementary Material**. The RNA-seq results showed that a large number of DEGs existed in six comparison pairs: W-S vs W-CK, KP-S vs W-CK, KP-S vs W-S, W-A vs W-CK, KP-A vs W-CK, and KP-A vs W-A (**Figure 8A**). A total of 5325 stress-responsive differentially expressed genes (DEGs) were identified in the W-S vs W-CK group, comprising 3085 upregulated and 2240 downregulated genes. Similarly, 8235 DEGs (5545 upregulated and 2690 downregulated) were detected in the W-S vs W-CK group, highlighting significant differences in stress responses between salinity and alkali stresses. Furthermore, 4125 DEGs (3103 upregulated and 1022 downregulated) were identified in the KP-S vs W-S group, while 1,971 DEGs (226 upregulated and 1745 downregulated) were found in the KP-A vs W-A group. This indicated a great difference in regulation pathways by KP-priming between salinity and alkali stress. There were 1403 DEGs among all salinity treatments and 1795 among all alkali treatments shared (**Figure 8B**), indicating a great similarity in salinity and alkalinity stresses responses between different treatments. The regulatory effects of exogenous regulators on plants and the self-regulatory mechanisms of plants exhibit a relatively consistent regulatory network facing the same stress. All of this showed that DEGs were likely necessary for the mechanism of KP-induced tolerance to salinity and alkalinity stresses in the rice seedling stage.

#### 3.5.2 Enrichment-based clustering of DEGs

**Figure 9:**
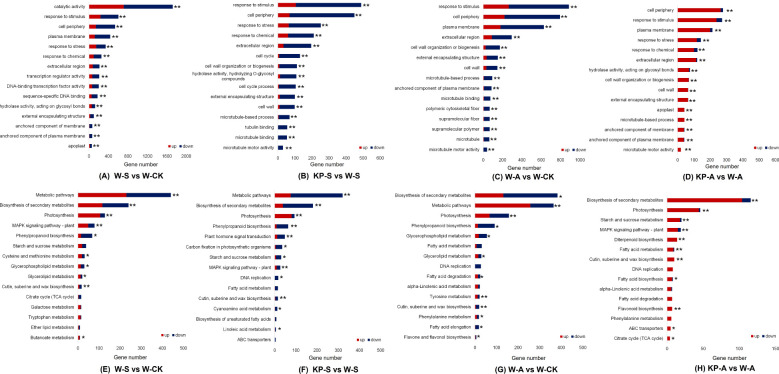
Kyoto encyclopedia of genes and genomes (KEGG) pathway enrichment analysis and enriched gene ontology (GO) annotation classification. There were 15 GO terms related to stress response **(A–D)**; 15 KEGG pathways commonly identified in different treatments of rice seedling leaves associated with stress response **(E–H)**. FDR, false discovery rate. ^*^ and ^**^ indicate significantly enriched at FDR < 0.05 and FDR < 0.01, respectively. W-CK, Distilled water priming + distilled water culture; W-S, Distilled water priming + 100 mmol/L NaCl culture; KP-S, KP-priming + 100 mmol/L NaCl culture; W-A, Distilled water priming + 10 mmol/L Na_2_CO_3_ culture; KP-A, KP-priming + 10 mmol/L Na_2_CO_3_ culture.

To explore the functional roles of the DEGs in response to stress conditions or exogenous KP-priming, we conducted GO and KEGG enrichment analyses on the DEGs identified in the comparisons of W-S vs W-CK, W-A vs W-CK, KP-S vs W-S, and KP-A vs W-A. The GO terms “response to stress,” “response to stimulus,” and “response to chemical” were significantly enriched for both treatments (except for W-A vs W-CK) (**Figures 9A–D**). We selected 15 commonly enriched pathways in both treatments shown in **Figures 9E–H**. Under salinity stress, both W-S vs W-CK and KP-S vs W-S exhibited a commonality of seven significantly enriched pathways: cutin, suberin, and wax biosynthesis; starch and sucrose metabolism; phenylpropanoid biosynthesis; MAPK signaling pathway, involving plant; photosynthesis; biosynthesis of secondary metabolites; and metabolic pathways (**Figures 9E, F**). Under alkalinity stress, both W-A vs W-CK and KP-A vs W-A exhibited a commonality of eight significantly enriched pathways: phenylalanine metabolism; cutin, suberin, and wax biosynthesis; alpha-linolenic acid metabolism; fatty acid degradation; DNA replication; fatty acid metabolism; photosynthesis; and biosynthesis of secondary metabolites (**Figures 9G, H**). It is noteworthy that photosynthesis was shared by these four comparisons. Overall, these results indicated that DEGs related to photosynthesis in rice seedling leaves may be regulated by KP-priming for the response to salinity and alkalinity stresses.

#### 3.5.3 Effects of KP-priming on leaf photosynthetic capacity under abiotic stress

In the comparisons of W-S vs W-CK, KP-S vs W-S, W-A vs W-CK, and KP-A vs W-A, 27 DEGs that were regulated by both stress conditions and KP-priming were identified. We hypothesized that these transcriptomic changes might play a role in driving the observed differences in leaf photosynthetic capacity among rice seedlings under salinity and alkalinity stresses (**Supplementary Table S4**). Based on our transcriptome data, several differentially expressed genes (DEGs) were identified and screened. Previous studies have demonstrated that the upregulation or downregulation of these genes can enhance plant tolerance to stress conditions. Overexpression of *OsRBCS5* increased the Rubisco content in rice leaves (Ogawa et al., 2012). Overexpression of *PGR5* helped to maintain the redox homeostasis in chloroplasts of rice leaves under the stress conditions (Nishikawa et al., 2012). The overexpression of *Se5* resulted in higher tolerance to oxidative stress (Ogawa et al., 2012). The phenotypes of plants overexpressing particular genes, *OsPORA* (Li et al., 2019), *GRA78* (Wang et al., 2019), *OsLhcb7* (Liang et al., 2022), and *OsPS1-F* (Ramamoorthy et al., 2018), were consistent with the higher chlorophyll content phenotype observed in rice leaves. All of the above genes were selected (P < 0.001). We hypothesized that these gene expressions might be directly or indirectly modulated by KP-priming, thereby mitigating the detrimental effects of salinity and alkalinity stress on photosynthesis in rice seedlings. Taken together, the gene expression results in different treatments were shown as **Table 3** and **Supplementary Figure S2**. Compared to W-CK, the expressions of *OsRBCS5, OsLhcb7, PGR5, OsPS1-F, OsPORA*, and *GRA78* were down-regulated in W-S and W-A treatments. However, the expressions of *OsRBCS5, OsLhcb7, PGR5, OsPS1-F, Se5, OsPORA*, and *GRA78* were up-regulated in KP-S compared to W-S, as well as up-regulated in KP-A compared to W-A. All of which strongly indicated that salinity and alkalinity stresses hindered photosynthesis through down-regulating the key photosynthetic genes, but KP-priming enhanced stress tolerance and seedlings growth through up-regulating genes.

**Table 3:** The DEGs related to photosynthesis in rice seedling leaves treated with KP-priming under salinity and alkalinity stresses.

Gene id	Gene name	Gene function	Up/down			
			W-S/W-CK	KP-S/W-S	W-A/W-CK	KP-A/W-A
*Os12g0291400*	*OsRBCS5*	Rubisco biosynthesis	Down	Up	Down	Up
*Os09g0296800*	*OsLhcb7*	Chlorophyll biosynthesis	Down	Up	Down	Up
*Os08g0566600*	*PGR5*	Redox balance of chloroplasts	Down	Up	Down	Up
*Os03g0778100*	*OsPS1-F*	Photosynthetic electron transport	Down	Up	Down	Up
*Os06g0603000*	*Se5*	Heme oxygenase biosynthesis	Up	Up	Up	Up
*Os04g0678700*	*OsPORA*	Chlorophyll biosynthesis	Down	Up	Down	Up
*Os01g0814800*	*GRA78*	Chlorophyll biosynthesis	Down	Up	Down	Up

W-CK, Distilled water priming + distilled water culture; W-S, Distilled water priming + 100 mmol/L NaCl culture; KP-S, KP-priming + 100 mmol/L NaCl culture; W-A, Distilled water priming + 10 mmol/L Na_2_CO_3_ culture; KP-A, KP-priming + 10 mmol/L Na_2_CO_3_ culture.

## 4 Discussion

### 4.1 Effects of KP-priming on rice seedling growth

Salinity and alkalinity stresses are common, and adversely affect different rice growth stages: reducing germination rate and survival rate of the rice seedling stage (Khan et al., 1997), and decreasing yield due to decreases in primary root length and the number of lateral roots at the reproductive growth stage (Khan and Abdullah, 2003; Jiang et al., 2023). It is important to note that the establishment of rice seedlings plays a critical role in subsequent growth and development, and enhancing stress tolerance at the seedling stage is directly linked to improved stress resilience and yield stability in later stages. Roots are the first organs to sense stress and experience significant growth inhibition, which subsequently impedes the morphological development of the aboveground parts. Transcriptomics analysis revealed that 1286 DEGs including 526 upregulated and 760 downregulated were identified as responding to salt stress in rice roots (Xue et al., 2024), finally hinder root development and growth. We found that stresses decreased root morphological indexes including root length, surface area, average diameter, and volume by 8.75–52.41% (salinity stress) and 17.32–46.33% (alkalinity stress) at all times of sampling the rice seedlings (**Table 1**). Previous study have demonstrated that the contact area between roots and soil as well as the photosynthetic capacity of the aboveground parts, are the most critical factors influencing the biomass of plant seedlings, ultimately determining their tolerance to saline-alkali stress (An et al., 2021). In this study, salinity and alkali stress severely inhibited the normal growth of rice seedlings, with significant reductions in plant height and evident leaf yellowing from 7 DAS (**Figure 2**), causing a reduction in plant height by 44.44–50.44% in salinity stress and by 49.83–59.04% in alkalinity stress, eventually leading to a significant reduction in total biomass by 60.18% (salinity stress) and 63.39% (alkalinity stress) at 21 DAS (**Figure 3**). The KP-priming significantly promoted growth of rice seedlings under both salinity and alkalinity stresses (**Figure 2**), with enhanced aboveground and underground growth (**Table 1**, **Figure 3**). This is consistent with the results of He et al. (2022) showing that oligosaccharides could alleviate cold stress by increasing aboveground biomass and improving phenotype of the root system in tomato seedlings. The regulatory mechanisms of KP-priming on rice seedlings were further analyzed and compared with findings from our previous studies, which demonstrated that foliar application of mixed-oligosaccharides on strawberry and cucumber seedlings significantly alleviated aboveground senescence and enhanced root vitality and morphology during productive growth stage (Xu et al., 2024). These results collectively indicated that foliar application of KP-priming positively influences the transport of photosynthates to the roots by enhancing photosynthesis in the aboveground parts, thereby promoting coordinated root-shoot growth. In this study, KP-priming of salt- and alkali-stressed seedlings resulted in improved growth conditions at 21 DAS compared to the initial stress stage (**Figure 2**), indicating that oligosaccharide molecules can directly and rapidly interact with leaf cells due to their high biocompatibility (Rabea et al., 2003). The progressive increases in the number of spray applications further alleviated the inhibitory effects of salinity and alkali stress on rice seedling growth.

### 4.2 Effects of KP-priming on rice seedling leaf cell membrane stability

Yin et al. (2024) revealed that metabolic pathways related to antioxidant responses and osmotic balance were crucial for salt-stress tolerance. Positive turgor pressure helps cells maintain normal morphology and function, and is essential for intra- and extra-cellular substance exchange. Osmotic regulation and cell membrane stability are important for turgor pressure (Luo et al., 2022). Osmotic regulation is the basic response of cells when exposed to osmotic stress, which is mainly realized by regulating the content of intracellular osmotic regulation substances, such as soluble sugar in this paper (**Figure 4**). Typically, a stronger osmotic regulation capacity reflects greater tolerance to osmotic stress. In the study, soluble sugar content was significantly increased by 11.76% (7 DAS) in KP-S compared to W-S, and by 5.70% (7 DAS) in KP-A compared to W-A, indicating that KP-priming could enhance tolerance to osmotic stress by increasing the soluble sugar content in cells. Cell membrane stability is essential for survival and normal cellular function; under environmental stress, excessive ROS and MDA are generated, causing lipid membrane peroxidation and increased membrane permeability (Zaidi et al., 2019). In our study, when rice seedlings were subjected to salinity and alkali stress, the MDA, O_2_ **^·^** ^−^, and H_2_O_2_ were significantly elevated in leaves (**Figure 4**), and a large amount of ROS was generated through the MAPK signaling pathway, which acted as a messenger in response to environmental stress (**Figures 9E–H**). Nevertheless, the KP-S and KP-A treatments markedly diminished the levels of MDA and H_2_O_2_ in the foliage throughout the entire duration, signifying that KP-priming substantially mitigated the oxidative harm inflicted by saline and alkalinity stresses on rice seedling leaves. It is noteworthy that the O_2_ **^·^** ^−^ content in rice seedling leaves treated with KP-priming was significantly higher than for W-priming under stresses, even higher than that of W-CK. On the one hand, reactive oxygen species (ROS) in appropriate concentrations serve as signaling molecules that play a role in the normal growth and development of cells, as well as in their adaptation to environmental conditions (Li and Kim, 2022). On the other hand, the application of KP-priming facilitates the acceleration of sugar metabolism in leaves, and the consequent surge in ATP synthesis results in an elevation of ROS levels (Yang et al., 2024). Consequently, the relationship between cell membrane stability and ROS is intricate; while an excessive accumulation of ROS can detrimentally impact the cell membrane, a balanced level of ROS generated by cells as a defensive mechanism against stress contributes to the preservation of cellular integrity (Li and Kim, 2022). The antioxidant system consisting of antioxidant enzymes (e.g. SOD, POD and CAT) can effectively reduce the ROS content in plants (Zhou et al., 2021; Lu et al., 2022). In this study, KP-priming significantly increased SOD, POD, and CAT activities in rice seedling leaves (**Figure 5**), consistent with our previous study on promotion of rice seed germination by KP-priming immersion through improving the antioxidant capacity (Yang et al., 2024). Compared to W-priming, overexpression of genes *PGR5* and *Se5* was found in KP-primed stress seedlings (**Table 3**). Previous studies confirmed that *PGR5* and *Se5* overexpression contributes to intracellular redox homeostasis (Nishikawa et al., 2012) and high tolerance to oxidative stress (Ogawa et al., 2012). It was deduced that KP-priming has the capacity to sustain cellular oxidative equilibrium through the modulation of *PGR5* and *Se5* gene expression. In summary, our preliminary findings suggested that KP-priming enhanced the tolerance of rice to salinity and alkalinity stresses by triggering diverse metabolic pathways that shield the seedlings from oxidative harm. This is of considerable importance for addressing the issue of low seedling survival rates indirect-seeded rice cultivation, which is often exacerbated by the presence of salinity and alkalinity stresses in field conditions.

### 4.3 Effects of KP-priming on rice seedling leaf photosynthesis

Seedlings exhibit notable net photosynthesis subsequent to the elongation of their first leaf, marking the shift from heterotrophic to autotrophic development in rice. The carbohydrates synthesized through photosynthesis serve as the primary energy reservoir for seedlings establishment, while the downward translocation of organic matter significantly contributes to root development. Consequently, photosynthetic efficiency is a pivotal determinant in the growth and development of seedlings (Xu et al., 2024). Treatment comparisons W-S vs W-CK, KP-S vs W-S, W-A vs W-CK, and KP-A vs W-A exhibited significant enrichment in photosynthesis (**Figures 9A–D**). This indicates that KP-priming potentially enhances the accumulation of leaf starch and soluble sugars by augmenting photosynthetic efficiency in the leaves thereby supplying essential energy substrates to support plant growth and development. Huo et al. (2024) used combined transcriptomic and metabolomic data confirming that Choline Chloride enhances rice salt tolerance by activating distinct transcriptional cascades and phytohormone signaling, along with multiple antioxidants and unique metabolic pathways, which were consistent with our study (**Figure 9**). Stomata serve as the primary gateways for gas exchange during photosynthesis and respiration, and they play a critical role in stress responses. For instance, they can modulate the proportions of completely open, partially open, and completely closed stomata to mitigate damage caused by water loss under external stress conditions (Luo et al., 2022). In our study, salinity and alkali stresses caused individual stomatal size of rice seedling leaves at 14 DAS to be smaller (**Figure 6**): stomatal length was significantly reduced by 23.13% under salinity stress; and stomatal number, length, and perimeter under alkali stress were significantly reduced by 33.33%, 16.11%, and 12.68%, respectively, representing normal physiological responses of plants when facing exogenous stress. These responses indicated that salinity and alkali stress caused physiological drought in leaves, and that leaves mitigated water loss by narrowing stomata. However, KP-priming significantly increased the number and size of stomata (**Table 2**), suggesting that KP-priming could alleviate the adverse effects of osmotic stress on leaves, thus maintaining normal stomatal size conducive to gas exchange and promoting high photosynthetic efficiency. The Chl a and Chl b are important photosynthetic pigments with important roles in chloroplasts to capture light energy and facilitate biochemical reactions. Salinity and alkali stress can significantly reduce the leaf chlorophyll content (Wang et al., 2021). In this study, after salinity and alkali stress, rice seedling leaves showed yellowing (**Figure 2**), and the corresponding Chl a and Chl b contents were significantly reduced, while KP-priming significantly increased the photosynthetic pigment contents of seedling leaves (**Figure 7**). Transcriptome data also revealed that genes *Se5*, *OsPORA*, *GRA78*, *OsLhcb7*, and *OsPS1-F* were upregulated in rice leaves when treated with KP-priming under salinity and alkalinity stresses compared to W-priming; it was inferred that KP-priming could increase the chlorophyll content of rice leaves by regulating expression of these genes (Ramamoorthy et al., 2018; Li et al., 2019; Wang et al., 2019; Liang et al., 2022). Chlorophyll fluorescence serves as a precise indicator of photosynthetic dynamics, capturing variations in processes such as light absorption, excitation energy distribution, and electron transport (Valkama et al., 2003). Moreover, the involved parameters are not only used to evaluate the PSII intensity of reaction, (e.g. Fv/Fm is positively correlated with photosynthetic efficiency; Wang et al., 2016), but also can be used as reference indicators of stress tolerance when the plant is subjected to abiotic stress (He et al., 2022). Salinity and alkali stress can significantly reduce Fv/Fm of plant leaves (Dong et al., 2023), consistent with our results (**Figure 7**). Our previous research on mitigating natural senescence in strawberry and cucumber demonstrated that foliar application of mixed-oligosaccharides during the seedling stage significantly enhanced leaf Fv/Fm after flowering. This aligns with the current study’s findings, where KP-priming treatment significantly increased Fv/Fm in rice seedlings across all observed periods post-spraying. Transcriptomic analysis further disclosed that the expression levels of *OsRBCS5* was upregulated in seedling leaves in the KP-priming treated groups (**Table 3**). It was hypothesized that oligosaccharide molecules may regulate leaf Rubisco content by affecting expressions of *OsRBCS5* expression (Ogawa et al., 2012), which in turn affects the light-conversion capacity of leaves. However, He et al. (2022) found that spraying different oligosaccharides did not increase Fv/Fm in tomato seedlings after cold stress, suggesting interspecies differences in the regulatory effects of oligosaccharides on PSII, which are also influenced by the external environment. Thus, salinity and alkalinity stresses can markedly diminish the photosynthetic efficiency of the aerial parts of rice and negatively impact the growth of rice seedlings. The application of KP-priming notably mitigated the detrimental effects of salinity and alkalinity stresses on rice seedlings and preserved elevated leaf photosynthetic capacity by influencing diverse metabolic pathways within the leaves, enabling the plants to adapt to challenging environmental conditions. Moreover, further research is needed on the effects of KP-priming on root of rice seedlings with direct-seeded technology, as well as the regulatory effects on direct-seeding rice yield at maturity in field production.

## 5 Conclusion

**Figure 10:**
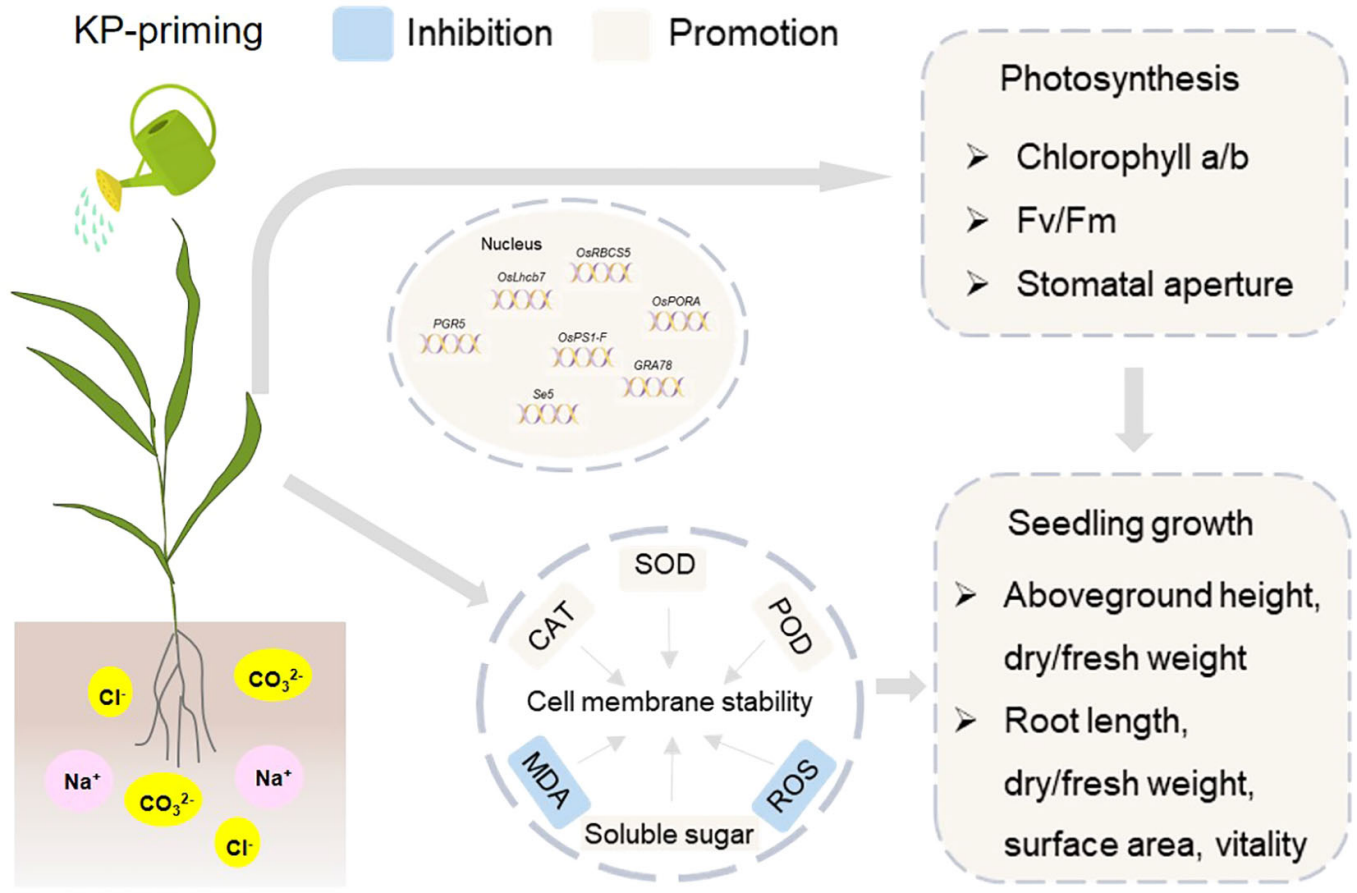
KP-Priming enhances rice seedling growth and stress tolerance via improved photosynthesis and cell membrane stability. Spraying KP-priming increased soluble sugar content in leaf cells, reduced ROS (reactive oxygen species) and MDA (malondialdehyde) levels, and enhanced antioxidant enzyme activities (e.g. SOD (superoxide dismutase), POD (peroxidase), and CAT (catalase)), effectively mitigating membrane oxidative damage and maintaining cellular stability. The up-regulation of key photosynthetic genes promoted the accumulation of Chl a and Chl b, and enhanced leaf Fv/Fm. Totally, the enhancements of photosynthesis and cell membrane stability synergistically promoted rice seedling shoot-root growth under salinity and alkalinity stresses, including the enhancements in shoot and root length, biomass accumulation, as well as root vitality and morphology.

Foliar application of exogenous KP-priming significantly mitigated the detrimental effects of salinity and alkalinity stresses on rice seedlings (**Figure 10**). The KP-priming could partially enhance rice seedling shoot-root growth under salinity and alkalinity stresses, including the enhancements in shoot and root length, biomass accumulation, as well as root vitality and morphology. The KP-priming increased soluble sugar content in leaf cells, reduced ROS and MDA levels, and enhanced antioxidant enzyme activities (e.g. SOD, POD, and CAT), effectively mitigating membrane oxidative damage and maintaining cellular stability. The DEGs were significantly enriched in pathways related to photosynthesis. The up-regulation of key photosynthetic genes promoted the accumulation of Chl a and Chl b, and enhanced leaf Fv/Fm. Our study suggested that KP-priming has potential as a novel plant growth regulator to improve tolerance of rice seedlings to salinity and alkalinity stresses, which lays the foundation for development of environmentally friendly and cost-effective strategies aimed at optimizing the utilization of saline-alkali land. Additionally, the findings will provide technical support for the efficient production of direct-seeded rice in the saline-alkali regions of Western China and contribute to ensuring regional food security.

## Data Availability

The RNA-Seq data described in this study can be found in NCBI, PRJNA1227679.
