# Supplementary material for: Transcriptome analysis reveals the mechanism of mixed oligosaccharides in the response of rice seedlings to abiotic stresses
Source: Front Plant Sci. 2025 Apr 28;16:1546679. doi: 10.3389/fpls.2025.1546679 (PMC12066455; doi:10.3389/fpls.2025.1546679)
Supplement: Supplementary file 1 [file DataSheet1.zip › Supplementary Figures and Tables.docx]

**Supplementary material**

Table S1 Characteristics of oligosaccharide materials

| Materials | Molecular formula | Molecular weight | pH | Source |
| --- | --- | --- | --- | --- |
| Cello-oligosaccharide | C_6_H_11_O_6_(C_6_H_10_O_5_)_4_ | 827 | 6.3 | Organic |
| Xylooligosaccharide | C_5_H_9_O_5_(C_5_H_9_O_4_)_3.1_ | 561.3 | 6.0 | Organic |
| Chitosan oligosaccharide | C_8_H_14_NO_6_(C_8_H_13_NO_5_)_4.6_ | 1159.4 | 6.4 | Organic |

Table S2 The detailed contents in nutrient solutions

| Components | Concentration (mg/L) | Components | Concentration (mg/L) |
| --- | --- | --- | --- |
| (NH_4_)_2_SO_4_ | 48.2 | H_2_MoO_4_**·**H_2_O | 0.09 |
| KH_2_PO_4_ | 24.8 | H_3_BO_3_ | 2.86 |
| KNO_3_ | 18.5 | ZnSO_4_**·**7H_2_O | 0.22 |
| K_2_SO_4_ | 15.9 | CuSO_4_**·**5H_2_O | 0.08 |
| MgSO_4_**·**7H_2_O | 135.1 | Na_2_EDTA | 7.45 |
| Ca(NO_3_)_2_**·**4H_2_O | 86.4 | FeSO_4_**·**7H_2_O | 5.57 |
| MnCl_2_**·**4H_2_О | 1.81 | Na_2_SiO_3_**·**9H_2_O | 200 |

Table S3 The primers used for qPCR

| Gene | Forward primer (5’ to 3’) | Reverse primer (5’ to 3’) |
| --- | --- | --- |
| *OsRBCS5* | GATTTCTCTGCGCAGGTGTG | GGAGACCTGTGGTTCTCACG |
| *OsLhcb7* | TTGTCAACAGTCACCTCACG | AATCCCGCAGTACCTTGCAT |
| *PGR5* | GCAGGTACGCACGTCTACTC | TCGATGGCCACAAGTCACTC |
| *OsPS1-F* | TATGGCCATGCACCATCACAT | CTCCACTGCCAAATCAACTGC |
| *Se5* | GAGCTCTATTTGCCACGCCA | CCAGGCAATGGTCCTTCTCC |
| *OsPORA* | TGCTCTCTCTGTCCCCAAGA | ACCCTGATCAGTCGTCACAC |
| *GRA78* | TGCGGTGAGAGCTTTTATCT | CCTCATTCTTCGGACCGAGT |

Table S4 The DEPs related to photosynthesis in rice seedling leaves with different treatment under salinity and alkalinity stresses

| Gene ID | Gene name | Gene description |
| --- | --- | --- |
| *Os05g0549100* |  | Similar to serine/threonine-protein kinase SNT7. (Os05t0549100-01); Similar to Serine/threonine-protein kinase SNT7, chloroplast precursor (EC 2.7.1.37) (Stt7 homolog). (Os05t0549100-02) |
| *Os12g0291400* | *OsRBCS5* | Similar to Petunia ribulose 1,5-bisphosphate carboxylase small subunit mRNA (clone pSSU 51), partial cds. (Fragment). (Os12t0291400-01) |
| *Os02g0152400* | *OsRBCS1* | Rubisco small subunit 1, Reguration of Rubisco catalytic activity (Os02t0152400-01) |
| *Os02g0714200* | *OsPFP1α1* | Similar to Pyrophosphate--fructose 6-phosphate 1-phosphotransferase alpha subunit (EC 2.7.1.90) (PFP) (6-phosphofructokinase, pyrophosphate dependent) (Pyrophosphate-dependent 6-phosphofructose-1-kinase) (PPi-PFK). (Os02t0714200-01) |
| *Os09g0296800* | *OsLhcb7* | Chlorophyll A-B binding protein family protein. (Os09t0296800-01) |
| *Os07g0147550* | *OsPsbR2* | Similar to Photosystem II 10 kDa polypeptide, chloroplast. (Os07t0147550-00) |
| *Os08g0566600* | *PGR5* | Similar to PGR5. (Os08t0566600-01) |
| *Os03g0190100* |  | UbiA prenyltransferase family protein. (Os03t0190100-01) |
| *Os04g0690800* | *psbS2* | 22-kDa Photosystem II protein, Photoprotection (Os04t0690800-01) |
| *Os03g0778100* | *OsPS1-F* | Similar to Photosystem-1 F subunit. (Os03t0778100-01); Similar to Photosystem-1 F subunit. (Os03t0778100-02); Similar to Photosystem-1 F subunit. (Os03t0778100-03) |
| *Os06g0603000* | *Se5* | Heme-oxygenase, Phytochrome chromophore biosynthesis, Nitric oxide- and auxin-induced lateral root formation (Os06t0603000-01) |
| *Os07g0558500* |  | Inositol phosphatase-like protein. (Os07t0558500-01); Similar to Protein THYLAKOID FORMATION1, chloroplastic. (Os07t0558500-02) |
| *Os04g0678700* | *OsPORA* | Similar to Protochlorophyllide reductase (EC 1.3.1.33) (PCR) (NADPH- protochlorophyllide oxidoreductase) (POR) (Fragment). (Os04t0678700-01) |
| *Os06g0229000* |  | Similar to FtsH protease (VAR2) (Zinc dependent protease). (Os06t0229000-01) |
| *Os06g0669400* |  | Similar to Cell division protease ftsH homolog 2, chloroplastic. (Os06t0669400-01); Similar to Cell division protease ftsH homolog 2, chloroplastic. (Os06t0669400-02); Similar to ATP-dependent zinc metalloprotease FTSH 2, chloroplastic. (Os06t0669400-03) |
| *Os06g0725900* |  | Similar to Cell division protein ftsH homolog, chloroplast precursor (EC 3.4.24.-) (DS9). (Os06t0725900-01); Similar to cDNA clone:J013001F19, full insert sequence. (Os06t0725900-02) |
| *Os05g0405000* | *flo4* | Orthophosphate dikinase precursor (EC 2.7.9.1). (Os05t0405000-01); Similar to Isoform 2 of Pyruvate, phosphate dikinase 1, chloroplastic. (Os05t0405000-02) |
| *Os01g0600900* |  | Chlorophyll a-b binding protein 2, chloroplast precursor (LHCII type I CAB-2) (LHCP). (Os01t0600900-02); Similar to Chlorophyll a-b binding protein 2, chloroplastic. (Os01t0600900-03) |
| *Os01g0720500* |  | Similar to Type I chlorophyll a/b-binding protein b (Fragment). (Os01t0720500-01); Non-protein coding transcript. (Os01t0720500-02) |
| *Os01g0814800* | *GRA78* | Similar to Cysteine synthase, chloroplast precursor (EC 2.5.1.47) (O-acetylserine sulfhydrylase) (O-acetylserine (Thiol)-lyase) (CSase B) (CS-B) (OAS-TL B). (Os01t0814800-01) |
| *Os04g0434900* |  | Similar to OSIGBa0102D10.5 protein. (Os04t0434900-00) |
| *Os04g0522800* |  | Photosystem II oxygen evolving complex protein PsbQ family protein. (Os04t0522800-01) |
| *Os04g0528800* |  | Similar to Ferredoxin-thioredoxin reductase, variable chain (FTR-V) (Ferredoxin- thioredoxin reductase subunit A) (FTR-A). (Os04t0528800-01); Similar to OSIGBa0115K01-H0319F09.23 protein. (Os04t0528800-02) |
| *Os05g0110800* |  | Conserved hypothetical protein. (Os05t0110800-01); Similar to predicted protein. (Os05t0110800-02) |
| *Os06g0229066* |  | Similar to ATP-dependent zinc metalloprotease FTSH 6, chloroplastic. (Os06t0229066-00) |
| *Os07g0567400* |  | Similar to Cytochrome c6. (Os07t0567400-01) |
| *Os11g0216300* | *OsABC1-13* | ABC-1 domain containing protein. (Os11t0216300-01); Similar to ABC1 family protein, expressed. (Os11t0216300-02) |

Figure S1. The PCA, volcano plots, and heat map images

Figure S2. qRT-PCR verification of expression levels of genes

*OsRBCS5: small subunit of Rubisco (Os12g0291400); OsLhcb7: light-harvesting chlorophyll a/b-binding (LHC) protein (Os09g0296800); PGR5: proton gradient regulation 5 (Os08g0566600); OsPS1-F: photosystem1-F subunit (Os03g0778100); Se5: photoperiod-sensitivity-5 (Os06g0603000); OsPORA: NADPH: protochlorophyllide oxidoreductase A (Os04g0678700); GRA78: S-sulfocysteine synthase; green-revertible albino mutant gra78 (Os01g0814800).* The error bars indicate standard deviations derived from three independent biological experiments. Significant differences were determined by t-test. *P < 0.05, **P < 0.01.
